# Structural ensemble of a glutamate transporter homologue in lipid nanodisc environment

**DOI:** 10.1038/s41467-020-14834-8

**Published:** 2020-02-21

**Authors:** Valentina Arkhipova, Albert Guskov, Dirk J. Slotboom

**Affiliations:** 1 Groningen Biomolecular Sciences and Biotechnology Institute (GBB), University of Groningen, Nijenborgh 4, 9747AG Groningen, The Netherlands; 2 Moscow Institute of Physics and Technology, Dolgoprudny, Russia; 3 Zernike Institute for Advanced Materials, University of Groningen, Nijenborgh 4, 9747AG Groningen, The Netherlands

**Keywords:** Membrane proteins, Cryoelectron microscopy, Cryoelectron microscopy

## Abstract

Glutamate transporters are cation-coupled secondary active membrane transporters that clear the neurotransmitter L-glutamate from the synaptic cleft. These transporters are homotrimers, with each protomer functioning independently by an elevator-type mechanism, in which a mobile transport domain alternates between inward- and outward-oriented states. Using single-particle cryo-EM we have determined five structures of the glutamate transporter homologue Glt_Tk_, a Na^+^- L-aspartate symporter, embedded in lipid nanodiscs. Dependent on the substrate concentrations used, the protomers of the trimer adopt a variety of asymmetrical conformations, consistent with the independent movement. Six of the 15 resolved protomers are in a hitherto elusive state of the transport cycle in which the inward-facing transporters are loaded with Na^+^ ions. These structures explain how substrate-leakage is prevented – a strict requirement for coupled transport. The belt protein of the lipid nanodiscs bends around the inward oriented protomers, suggesting that membrane deformations occur during transport.

## Abstract (web summary)

Glutamate transporters are membrane transporters that clear the neurotransmitter L-glutamate from the synaptic cleft via a so-called elevator mechanism. Here the authors present five cryo-EM structures of the transporter homologue Glt_Tk_, which explain how substrate leakage is prevented.

## Introduction

In humans, glutamate transporters (also known as excitatory amino acid transporters, EAATs) take up the neurotransmitter l-glutamate from the synaptic environment, which is necessary to sustain efficient neuronal communication and prevent neurotoxicity^1–3^. EAATs couple uptake of one substrate molecule to the transport of three sodium ions and one proton and counter-transport of one potassium ion^4–6^. Prokaryotic glutamate transporters homologs share high structural similarity with EAATs and neutral amino acid transporters ASCTs, which all belong to the solute carrier family 1 (SLC1A)^7^. Structural studies of the archaeal homologs Glt_Ph_ from *Pyrococcus horikoshii* and Glt_Tk_ from *Thermococcus kodakarensis* have provided the structural basis for understanding of the transport mechanism^8–13^. Both Glt_Ph_ and Glt_Tk_ couple uptake of one aspartate molecule to symport of three sodium ions^12,14^.

Glutamate transporters and their archaeal homologs are homotrimers, in which each protomer consists of a rigid scaffold domain involved in oligomerization and anchoring of the protein in the membrane, and a mobile transport domain that binds the substrate and cations and transports its cargo in an elevator-like motion across the membrane^10^. During movement of the transport domain, the substrate binding site is occluded from the solvent by two pseudo-symmetrical helical hairpins HP1 and HP2. The latter hairpin was shown to work as both an extracellular^9,15,16^ and an intracellular^17^ gate.

Mutagenesis studies^18–20^, single molecule fluorescence resonance energy transfer (smFRET)^21,22^, high-speed atomic force microscopy studies (HS-AFM)^23^, and molecular dynamics(MD) simulations^24,25^ strongly indicate that the transport domains of the three protomers move independently, hence should frequently visit asymmetric states during turnover conditions. However, extensive structural studies of glutamate transporters have revealed almost exclusively symmetrical arrangements of transport domains either in outward or inward states^9,10,12,17,26–28^. The only asymmetric state observed to date is in the crystal structure of a Glt_Ph_ mutant^29^. The discrepancy between the structural information on these trimeric proteins and the data from dynamics studies may originate from the detergent-solubilized state, which was used for determination of all reported structures, and that is a very different environment compared to the native membrane. Reconstitution of proteins into lipid nanodiscs formed by scaffold proteins allows to better mimic the lipid bilayer environment and may help to avoid some of the possible detergent artefacts^30^.

Here we report cryo-EM structures of the glutamate transporter homolog Glt_Tk_ in nanodiscs, revealing a variety of asymmetric arrangements of the transport domains. In addition, previously undetected conformations of the individual protomers provide structural insight in the elevator mechanism.

## Results

### Cryo-EM structures of Glt_Tk_ in nanodiscs

**Fig. 1:**
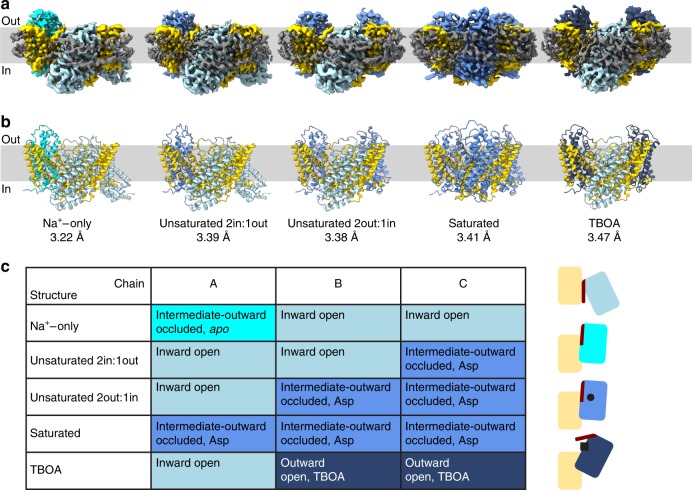
Conformational states of the trimeric Glt_Tk_. **a**, **b** Volume and cartoon representation of five cryo-EM structures of Glt_Tk_ in MSP2N2 nanodiscs obtained in the absence of substrate (Na^+^-only), in presence of L-aspartate (unsaturated 2in:1out, unsaturated 1in:2out, saturated) or in the presence of DL-TBOA. The approximate position of the lipid bilayer is represented by the light gray bar, with indication of sides of the membrane (*in* and *out*). **a** The cryo-EM density for the MSP2N2 belt is shown in dark gray. The transport domains of the individual protomers are present in four different conformations: inward open (steel blue), intermediate-outward occluded *apo* (cyan), intermediate-outward occluded Asp (cornflower blue), outward-open TBOA (dark blue). The scaffold domains are shown in yellow. **c** Table summarizing conformations of Glt_Tk_ protomers. Schematic representation of the conformations on the right in the same colors as in **a**, **b**, with indication of l-aspartate (black circle), DL-TBOA (black square) and HP2 (dark red stick).

Purified Glt_Tk_ was reconstituted into nanodiscs using the MSP2N2 scaffold protein^31^ and a mixture of *E.coli* polar lipids and egg PC (3:1 (w/w)), a lipid composition that supports robust transport activity of the protein in proteoliposomes^11,32^. Glt_Tk_-nanodiscs were concentrated to 4.5–9.0 µM, and supplemented with 300 mM Na^+^. To these preparations we added either nothing (Na^+^-only condition), or different concentrations of l-aspartate, or the competitive inhibitor dl-threo-beta-benzyloxyaspartate (TBOA inhibited). The preparations were analyzed by single particle cryo-electron microscopy with the aim to obtain structural insight in the conformational ensemble under turnover and stalled conditions (See Table 1 and Supplementary Fig. 1 for the cryo-EM workflows). We solved five structures of Glt_Tk_ (with resolutions of 3.2–3.5 Å, Supplementary Fig. 2), each with the protomers in a different trimeric arrangement (Fig. 1). In the collective set of structures, the 15 individual protomers adopted four different conformations (Fig. 1), which are characterized by their position relative to the scaffold domain (inward, outward, or intermediate-outward), the accessibility of the aspartate binding site (open or occluded), and the presence of substrates (*apo*, Na^+^-only, *holo* (Asp), *holo* (TBOA)). With one exception (when the Glt_Tk_ binding sites were saturated with L-aspartate), the arrangements of the transport domains in the trimer are non-symmetrical.

**Table 1:** Cryo-EM data collection, refinement and validation.

	Na^+^-only	Unsaturated 2in:1out	Unsaturated 2out:1in	Saturated	TBOA
Data collection and processing					
Voltage (kV)	200	200	200	200	200
Electron exposure (e^−^/Å^2^)	53.3	53.3	53.3	53.3	53.3
Defocus range (μm)	−0.5 to −2.0	−0.5 to −2.0	−0.5 to −2.0	−0.5 to −2.0	−0.5 to −2.0
Pixel size (Å)	1.012	1.012	1.012	1.012	1.012
Symmetry imposed	C1	C1	C1	C3	C1
Initial dataset (# of particles)	1,193,046	348,173	348,173	444,955	578,728
Final dataset (# of particles)	88,525	59,666	72,313	65,762	132,917
Map resolution (Å) FSC_01.43_	3.22	3.39	3.38	3.41	3.47
Refinement					
Initial model used	PDB 5E9S	PDB 5E9S	PDB 5E9S	PDB 5E9S	PDB 5E9S
Map-sharpening *B* factor (Å^2^)	84.8	101.7	81.0	129.3	135.9
Model composition					
Nonhydrogen atoms	9564	9555	9552	9572	9547
Protein residues	1278	1281	1280	1281	1276
Ligands	–	1	2	3	2
Mean *B* factors (Å^2^)					
Protein	79.75	45.02	61.70	79.02	15.08
Ligand	–	49.56	64.96	74.38	36.52
R.m.s. deviations					
Bond lengths (Å)	0.008	0.006	0.007	0.003	0.004
Bond angles (°)	0.747	0.684	0.708	0.600	0.610
Validation					
MolProbity score	1.95	1.92	1.95	1.79	1.80
Clash score	10.98	9.83	10.65	10.27	10.91
Poor rotamers (%)	0.00	0.00	0.00	0.00	0.10
Ramachandran plot (%)					
Favored	94.26	94.03	93.87	96.23	96.38
Allowed	5.74	5.97	6.13	3.54	3.62
Outliers	0.00	0.00	0.00	0.24	0.00
Model to map fit CC	0.83	0.8	0.84	0.77	0.78

### Aspartate-free conditions

**Fig. 2:**
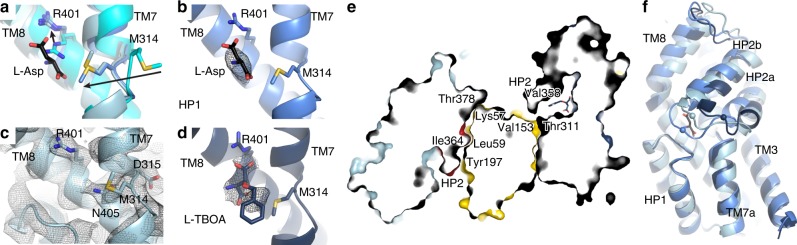
Glt_Tk_ substrate binding site and HP2 opening. **a** Overlay of *apo* (cyan), *holo* (Asp) (cornflower blue) and Na^+^-only states (light blue). The shift of Met314 and Arg401 is shown with arrows. **b** Cryo-EM density of l-aspartate (black mesh at 5σ). **c** Absence of the substrate in the inward open state (density is shown as gray mesh at 5σ). **d** Cryo-EM density of L-TBOA (black mesh at 4σ). **e** Slice through of TBOA-inhibited Glt_Tk_ structure (surface representation) showing an inward-oriented and outward-oriented protomer. Opening of HP2 on both sides of the membrane prevents movements of the transport domains. **f** Superposition (on HP1) of the transport domains in inward Na^+^-only, intermediate-outward *holo*-Asp and fully-outward TBOA-inhibited (dark blue) states. Opening of HP2 in the inward state (4.4 Å) and TBOA-inhibited state (10.4 Å) in comparison with the occluded state is measured using Cα of Val358 (shown as sphere). l-aspartate (black sticks) indicates position of the substrate-binding site.

Cryo-EM analysis of Glt_Tk_ in the presence of 300 mM Na^+^, but in the absence of the substrate l-aspartate showed an asymmetric trimer with two transport domains in an inward position, and the third one in an intermediate-outward position. The latter transport domain has an occluded binding site, with the HP2 gate closed (distance between the tips of HP1 and HP2 ~ 4.4 Å, Supplementary Table 1). We infer that this protomer is in the *apo* state, because the conformation of the transport domain is identical to that of the transport domains in the *apo*-crystal structure of Glt_Tk_ (PDB 5DWY, rmsd 0.534 Å)^12^. In the *apo*-state the binding sites of sodium ions are deformed, and the binding site for aspartate is non-existent because the conformation of the chain of Arg401 is incompatible with aspartate binding (Fig. 2a). Despite the similarity in conformation of the *apo*-transport domains in the cryo-EM and crystal structures, the position of the transport domain relative to the scaffold domain is different. In the cryo-EM structure, the transport domain is in an intermediate-outward position, whereas in the crystal structure (PDB 5DWY)^12^ it is fully outward. The position of the transport domain is roughly similar to that of an intermediate-outward domain observed in a crystal structure of Glt_Ph_ (PDB 3V8G, chain C^29^; rmsd 1.261 Å, Supplementary Fig. S3a), but whereas the Glt_Ph_ protomer was in a *holo* state (Na^+^-bound and Asp-bound), the Glt_Tk_ protomer is in the *apo* state.

In the two inward-oriented transport domains the gate formed by HP2 is open (distance between the tips of HP1 and HP2 ~ 9.3 Å, Supplementary Table 1), and the empty binding site for aspartate (Fig. 2c) is accessible to the aqueous solution. The conformation of the protomers strongly suggests that the inward-open protomers are in a Na^+^-bound state. This interpretation is based on the observation that two of the three binding sites for Na^+^ ions (Na1 and Na3), which are deformed in the *apo* protein^12^, are reshaped in the cryo-EM structure, consistent with bound Na^+^ ions. The presence of cryo-EM density in these sites further supports the interpretation that sodium ions are indeed bound, although the densities do not allow unequivocal assignment at the obtained resolution of 3.2 Å (Supplementary Fig. 4a). The Na2 site is not formed properly, because the open HP2 gate is incompatible with the Na2 site geometry (Supplementary Fig. 4b). A prominent indicator for the Na^+^-bound geometry at sites Na1 and Na3 is the conformation of the central unwound region of transmembrane helix 7 (TM7), around Met314. The sidechain of Met314 points away from the binding site in the *apo* state, as first shown in a crystal structure of Glt_Tk_^12^, and later also observed for Glt_Ph_^33^ (see also Fig. 2a), but is rotated over a distance of 9.7 Å in the bound state.

Na^+^ binding was shown previously to lead allosterically to formation of a high-affinity aspartate-binding site^11,12,34,35^. In the cryo-EM structure of the inward-oriented protomers, the side chain of Arg401, which adopts a conformation incompatible with aspartate binding in the *apo*-state, indeed has taken the position required for high-affinity L-aspartate binding (Fig. 2a). While an l-aspartate binding site is present in these protomers, it is unoccupied, consistent with a lack of cryo-EM density (Fig. 2c). The protomers are thus in a hitherto elusive Na^+^-only state.

The fact that we observe one protomer in the *apo* state and two in Na^+^-bound states, indicates that we did not manage to saturate all protomers with Na^+^ using a concentration of 300 mM Na^+^. This observation is consistent with reported *K*_d_ values for sodium binding to Glt_Ph_ (100–140 mM)^36^. Because of the sub-saturating Na^+^ concentration, Glt_Tk_ trimers with different ratios of *apo* and Na^+^-bound protomers were also expected in the dataset. Analysis of particles discarded during heterogeneous refinement for this dataset indeed revealed the presence of another asymmetric species with one inward-oriented and two outward-oriented transport domains, but this structure could be refined only up to 4.8 Å resolution. Possibly, the collection and analysis of more particles would allow the determination of this structure at higher resolution, as well as the detection of Glt_Tk_ in other states described by the binomial distribution of protomers over the *apo* and Na^+^-bound states, similar to what we will describe below for the substrate-unsaturated conditions.

### Fully loaded state

**Fig. 3:**
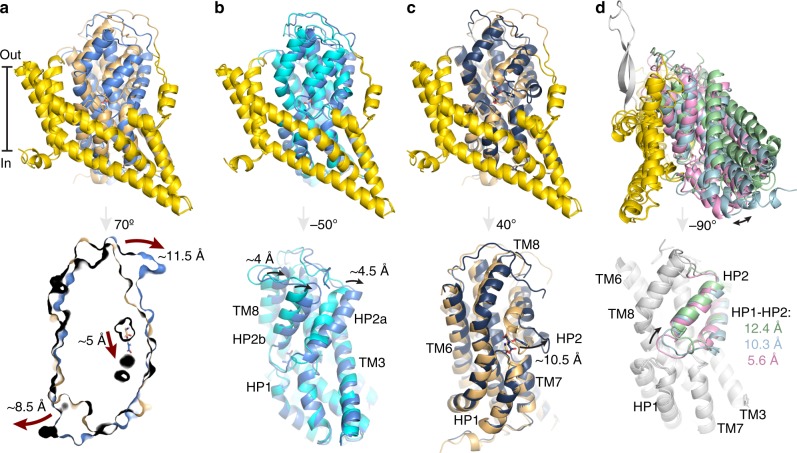
Conformational differences of the Glt_Tk_ protomers. **a** Comparison of the *holo* (Asp) Glt_Tk_ cryo-EM (cornflower blue) and crystal (PDB 5E9S, light orange)^12^ structures (superposition using scaffold domains (yellow)) demonstrates differences between the fully outward and intermediate outward states. The panel below shows a slice through the transport domains in surface representation with aspartate molecules shown as sticks. The arrows indicate the movement of the transport domain from the fully-outward to intermediate-outward position. **b** Superposition on the scaffold domains of the cryo-EM structures of Glt_Tk_- *holo* (Asp) (cornflower blue) and Glt_Tk_-*apo* (cyan), which are both in intermediate-outward conformations. The lower panel shows the transport domains rotated 50° relative to the upper panel to highlight structural differences. **c** Superposition on the scaffold domains of cryo-EM structure of Glt_Tk_-TBOA (dark blue) and crystal structure Glt_Tk_-Asp (PDB 5E9S, light orange). The lower panel shows the transport domains rotated 40° relative to the upper panel with an arrow indicating opening of the HP2 gate. **d** Superposition of the cryo-EM structure of Glt_Tk_-Na^+^-only (light blue), the cryo-EM structure of ASCT2 (PDB 6RVX, light green)^17^, and a crystal structure of Glt_Ph_-Asp in the inward-oriented state (PDB 3KBC, pink)^10^. The lower panel highlights opening of the HP2 gate in the inward-oriented state. Superpositions on TM2 and TM5 of the scaffold domain.

At the concentration of 300 mM Na^+^ used in the experiments presented here, the apparent K_d_ for l-aspartate is ~120 nM^32^, and therefore addition of 50 µM l-aspartate to 5.6 µM nanodiscs is expected to lead to substrate saturation. The cryo-EM structure of Glt_Tk_ in the *holo* (Asp) state is symmetrical with all three transport domains in an intermediate-outward position (Figs. 1, 3a). This position is similar to the ones found in a protomer of Glt_Ph_ (PDB 3V8G, chain C^29^; rmsd 0.965 Å, Supplementary Fig. 3b) and in the *apo* state described above, showing that the intermediate-outward orientation is visited by both the *holo* and *apo* transporters (Fig. 3b). In addition, the HP2 gates are closed in both cases, consistent with the ability to make elevator-type movements (Supplementary Table 1). Despite these similarities, there are also conspicuous differences. In the cryo-EM map of the *holo* (Asp) state, density for the amino acid substrate in the binding site is observed (Fig. 2b). Although the resolution is not high enough to unambiguously assign the density to aspartate, the positions of the binding residues are virtually the same as found in the crystal structure of Glt_Tk_ with l-aspartate bound (PDB 5E9S)^12^. The conformation of residues involved in binding of the three Na^+^-ions also differs between the *apo* and *holo* (Asp) states, with the correct binding site geometries only adopted in the latter. Finally, the C-terminal half of TM7, the helices of HP2 and the N-terminal half of TM8 in the *holo* (Asp) state are displaced away from the center of the trimer by ~4 Å (Fig. 3b), which is another previously observed difference between the *holo* and *apo* states of Glt_Tk_ and Glt_Ph_^11,12,33^.

### Substrate unsaturated conditions

Using a sub-saturating aspartate concentration, in which we aimed to occupy ~1/3rd of the aspartate binding sites in the nanodisc preparation, we were able to solve two distinct structures, with different asymmetric arrangements of the transporter domains (Fig. 1). Roughly equal amounts of particles were used for the two reconstructions (Supplementary Fig. 1c). The individual protomers in the two structures are either in the inward-open state, identical to the state observed in the Na^+^-only condition, or in the outward-intermediate state, identical to the ones observed under aspartate-saturated conditions. The difference between the two structures is the number of protomers in each state, with either two inward and one intermediate-outward (2 in:1 out), or one inward and two intermediate-outward (1 in:2 out) oriented protomers. Our data indicate that we visualized a part of the multinomial distribution of the transport domains over all different possible states. The frequency of particles found with “2 in:1 out” or “1 in:2 out” arrangement (89% of all particles) indicates that the inward-open and *holo* outward-intermediate states had the highest probabilities of occurrence under the substrate-unsaturated conditions. It is possible that more states and a more complete multinomial distribution could be resolved if many more particles were collected.

### TBOA-inhibited condition

In the four structures presented above, none of the protomers was in a fully outward orientation, which is remarkable, because this orientation is most frequently observed in crystal structures of both Glt_Ph_ and Glt_Tk_. To test whether this state could be visited in our nanodisc preparations, we determined a structure in the presence of 120 µM DL-TBOA. This bulky competitive inhibitor of aspartate transport sterically prevents the closure of the HP2 gate, and traps the transporter in an open state, which in the crystal structures of Glt_Ph_ is exclusively fully-outward (PDB 2NWW)^9^. Cryo-EM analysis of Glt_Tk_ in this condition shows an asymmetric trimer with one transport domain positioned in an inward-oriented state and two domains in a fully-outward-state. The structure of the inward-oriented protomer is identical to the ones described above, and is similarly interpreted as representing a Na^+^-only state. The fully-outward protomers have wide open HP2 gates (distance between the tips of HP1 and HP2 13.8–14.5 Å), and show density for bound TBOA (Figs. 2d, 3c). The structures of these protomers are similar to those of crystal structures of Glt_Ph_ in the presence of TBOA (rmsd 0.670 Å), and show that the fully-outward conformation is accessible in the nanodisc environment. This structure also reveals that the tip of HP1 of Glt_Tk_ moves as much as 24 Å across the lipid bilayer in the transition between inward and outward states (Supplementary Table 2). The asymmetric Glt_Tk_ structure with only two protomers occupied by the inhibitor suggests that the concentration of TBOA used was not saturating, which is consistent with the concentrations of Na^+^, DL-TBOA and protein used in the experiment, expected to lead to ~83% saturation.

### Lipids and membrane scaffold protein

We selected the MSP2N2 belt protein for reconstitution, because it is able to form nanodiscs with large diameter (15 nm)^37^, however our structures show that their diameter is only ~10–11 nm, and the belt wraps around the protein more tightly (Supplementary Fig. 5). Nonetheless, we deduce from the structures that the transport domains had the freedom to move between inward and outward positions while embedded in the nanodiscs. All nanodisc samples were prepared in an identical way in the absence of substrates. The observation that redistributions of the transport domains to different conformations occurred upon addition of l-aspartate or DL-TBOA to the Glt_Tk_ nanodiscs, shows that the belt protein did not prevent movement of the transport domains across the membrane.

**Fig. 4:**
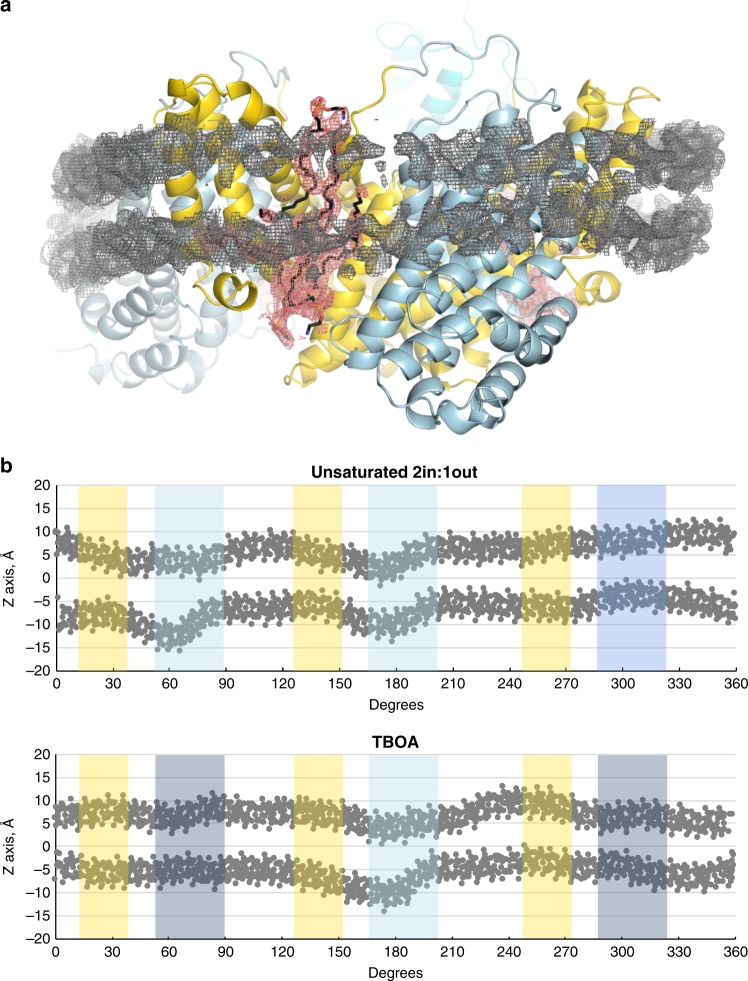
Lipids and membrane deformation. **a** MSP2N2 (gray mesh, 3.5 σ) and lipids (black sticks, salmon mesh, 3 σ) in the Glt_Tk_ structure determined in Na^+^-only conditions. Colors are the same as in Figs. 1–3. **b** Glt_Tk_ trimers were oriented with the *z*-axis along the pseudo three-fold axis of the scaffold domains, and the *y*-coordinates of the atoms from the modeled MSP2N2 protein were plotted as a function of the position along the 360° perimeter. The vertical colored bars indicate where the belt covers the scaffold (yellow) and transport domains (shades of blue as in Fig. 1c). Source data are provided as a Source Data file.

Cryo-EM densities resembling phospholipids in a bilayer-like arrangement were found in the crevices between the protomers of the Glt_Tk_ trimers, and indicate the position of the membrane (Fig. 4a). Since lipid-like densities were found only in these crevices, it was not possible to follow the shape of the lipid bilayer along the entire perimeter of the reconstituted protein. Therefore, we inspected the conformation of the MSP2N2 belt proteins, which were resolved in all structures obtained here. We used them as a guide to locate the position of the bilayer, also in places where lipid densities were not visible, and to estimate the extent of membrane deformations. We oriented the Glt_Tk_ trimers with the *z*-axis along the pseudo three-fold axis of the scaffold domains, and then plotted the *y*-coordinates of the atoms from the modeled MSP2N2 protein as a function of the perimeter position (Fig. 4b). A straight horizontal conformation of the belt protein indicates a planar lipid bilayer, whereas buckling of the belt protein is indicative of membrane deformation. The straightest conformation of the MSP2N2 protein was found around the protomers with outward oriented transport domains, whereas the largest extent of buckling was found around the inward-oriented protomers. Apparently, buckling of the belt proteins did not lead to strongly disfavored conformations of Glt_Tk_ in the nanodiscs, as in 6 out of the 15 protomers in the collective set of structures the transport domains are in inward positions. A recent molecular dynamics simulation on Glt_Ph_ also showed perturbations of the lipid bilayer around inward-oriented transport domains of Glt_Ph_^38^. The buckling of the belt proteins in our structures resembles these perturbations, indicating that the conformation of belt proteins provides at least an approximate position of the bilayer.

## Discussion

The collection of cryo-EM structures of nanodisc-reconstituted Glt_Tk_ presented here, reveals a variety of asymmetric protomer arrangements in the trimeric protein, consistent with a transport mechanism in which the protomers operate independently from each other, as shown by smFRET^21,22^, AFM^23^, MD simulations^24^, and mutagenesis studies^18^. The individual protomers were found in four different states, and cover the full trajectory between inward-open and outward-open states. Two of the elucidated states have not been observed previously: a Na^+^-only inward-open state, and an *apo*-occluded intermediate-outward state.

The Na^+^-only state provides insight in the mechanism by which substrate and Na^+^ leakage is prevented, and thus strict coupling between Na^+^ and aspartate transport is maintained. Leakage would occur if reorientation of the inward-open to the outward-open state were possible in the Na^+^-only state. The structure of the inward-open state presented here shows how this transition is prevented. The HP2 gate is open, and blocks movement of the transport domain. The same HP2 gate also opens in the outward-oriented state, captured here in the presence of TBOA, and prevents the transport domain from moving inward (Fig. 2e). In both cases, the tip and N-terminal helix of HP2 (HP2a) are displaced away from the aspartate binding site, and obstruct the movement of the transport domain along the scaffold (Fig. 2f). This mechanism, in which a single gate is used on both sides of the membrane resembles the one-gate elevator mechanism recently described for the neutral amino acid transporter ASCT2^17^. The extent of gate opening in inward-open Na^+^-only Glt_Tk_ (~9.3 Å distance between the tips of HP1 and HP2) differs from the opening in inward-open ASCT2, but is large enough for substrates to pass. Also, the position of the inward-oriented transport domains deviates somewhat between the structurally characterized proteins (Fig. 3d), which could be related to differences between detergent-solubilized and nanodisc-reconstituted proteins.

We had not anticipated to capture an inward-open state in the presence of only Na^+^, because for Glt_Ph_ it has been shown that the outward-facing state binds Na^+^ with three-fold higher affinity than the inward facing state^34^, and therefore the outward Na^+^-only state was expected to be favored. Similarly, in a high-speed AFM study of Glt_Ph_ the outward facing state was favored in the presence of 1 M Na^+^^23^. The fact that Glt_Tk_ was stable in the inward-open Na^+^-only state could be related to differences between detergent-solubilized protein (used for the Glt_Ph_ binding assays), bilayers with densely packed, reconstituted Glt_Ph_ on a mica support (used for the AFM study), and nanodisc reconstituted protein (used for Glt_Tk_ here), or *bona fide* differences between the two proteins. In living cells, the captured inward-open conformation of Glt_Tk_ would correspond to a state from which aspartate has been released following transport, and Na^+^ ions are on the verge of being released. It is unlikely to be a low energy state in vivo, because the intracellular Na^+^ concentration is low, and therefore dissociation of Na^+^ yielding the *apo* state will be favored. In the *apo*-state the HP2 gate can close, leading subsequently to reorientation of the transport domain to the intermediate-outward position, observed in *apo* structure presented here.

An intermediate-outward state is found in 7 of the 15 protomers from the five structures determined here, indicating that it is a low-energy conformation. A similar state was observed only for one protomer (Supplementary Fig. 3) in 34 available structures of Glt_Ph_^8–10,29,33,34,39–41^, Glt_Tk_^11,12,28^, EAAT1^26^, and ASCT2^17,27,42^. The prominent presence of the intermediate outward state in our cryo-EM structures suggests that it is favored in the membrane-environment, whereas detergent solution or crystal packing may favor the fully outward state. The outward-intermediate position of the transport domain was observed both in the fully loaded *holo*-(Asp)-state, and in the *apo*-state, suggesting that it is used both in the substrate-transport step and in the return of the empty carrier (Supplementary Fig. 6). It is not clear whether the binding site can become accessible by opening of the HP2 gate when the transport domain is in the intermediate-outward state, or that a transition to the fully outward state is needed for gate opening. The structures in the presence of TBOA suggest the latter. In vivo, the *apo*-intermediate state may not be long-lived, as excursions to the outward-open state in the presence of high external Na^+^ concentrations may stabilize a Na^+^-only state. We have been unable to capture the outward-open Na^+^-only state, because we could not introduce sidedness to the Na^+^ addition, and the inward-open Na^+^-only state of Glt_Tk_ in lipid nanodiscs is more stable at high Na^+^-concentration.

Despite the observation that Glt_Tk_ is tightly wrapped by and can potentially interact with the belt proteins, it can still bind the substrates and undergo large conformational changes. Nevertheless, the close proximity of the belt protein and Glt_Tk_ might affect the conformational ensemble and create artefacts similar to crystal contacts in protein crystals, or sub-optimal lipid compositions in bilayers^43^. Despite an increasing amount of cryo-EM structures of membrane proteins in nanodiscs, discussions on the tightness of the belt protein are very often omitted.

In conclusion, the collection of structures presented here provides insight into the conformational ensemble of a secondary active transporter in conditions where conformational changes related to transport take place, similar to what has been done for ATP-driven transporters (the ABC transporter TmrAB^44^ and the mitochondrial ATP synthase^45^). Our work reveals a rich diversity of previously undetected asymmetric states of Glt_Tk_, consistent with independent activity of the protomers. At the same time, our analysis shows that a lack of compartments, cation gradients and membrane voltage may obscure mechanistic details. Future developments in structural analysis of liposome-reconstituted proteins may open the way for structure determination under transporting conditions^46^.

## Methods

### Production and purification of MSP2N2

The gene encoding the membrane scaffold protein MSP2N2 with the His_7_-tag at the N-terminus cloned in the pET28a plasmid (Addgene) was expressed in *E.coli* BL21(DE3) cells (New England Biolabs). Cells were cultivated in LB medium in the presence of 50 µg/mL of kanamycin at 37 °C, 200 r.p.m. until the OD_600_ reached 0.8. Then expression was induced by addition of 0.1 mM IPTG and after 3.5 h cells were harvested by centrifugation (15 min, 6270 × *g*, 4 °C). Cells were resuspended in 40 mM Tris-HCl, pH 7.8, 300 mM NaCl and stored at −80 °C. For purification cells were thawed and supplemented with 100 µg/mL DNase A, 1 mM MgSO_4_ and 1 mM PMSF. Cells were passed through a cell disrupter at 4 °C and 25 kPsi (Constant Systems Ltd. Daventry UK). One percent of TritonX-100 was added to the lysate followed by stirring for 10 min at room temperature. After centrifugation at 30,000 × *g*, 30 min, 4 °C supernatant was supplemented with 20 mM Imidazole, pH 8.0, and MSP2N2 was purified using Ni-NTA resin. MSP2N2 was eluted in 40 mM Tris-HCl, pH 8.0, 300 mM NaCl, 500 mM Imidazole. To cleave off the His-tag, protein fractions were pooled, supplemented with 1 mM Na-EDTA and 1:40 w/w TEV (Sigma-Aldrich) and dialyzed against 20 mM Tris-HCl, pH 8.0, 100 mM NaCl, 0.5 mM EDTA, 0.5 mM DTT overnight at 4 °C using a Servapor® dialysis tubing. The cleaved protein was loaded onto Ni-NTA resin and collected in the flow-through fraction using 50 mM Tris-HCl, pH 8.0, 100 mM NaCl. Protein fractions were stored at −80 °C.

### Production and purification of *apo* Glt_Tk_

For production of C-terminally His_8_-tagged Glt_Tk_, *E. coli* MC1061 containing a pBAD24 derived plasmid were grown in LB medium with 100 µg/mL ampicillin at 37 °C, 200 r.p.m. When the OD_600_ reached 0.8 expression was induced with 0.05% l-arabinose for 3 h. Cells were harvested by centrifugation (15 min, 7400 × *g*, 4 °C) and resuspended in 20 mM Tris-HCl, pH 8.0. After breaking cells (25 kPsi, 5 °C, Constant Systems Ltd. Daventry UK) the membrane fraction was collected by ultracentrifugation of the supernatant (90 min, 193,360 × *g*, 4 °C), resuspended in 20 mM Tris-HCl, pH 8.0, and stored at −80 °C. To obtain *apo*-Glt_Tk_ Na^+^ was omitted from all buffers. An aliquot of membrane vesicles representing ~1.2 g cells was solubilized in 50 mM Tris-HCl, pH 8.0, 300 mM KCl, 1% n-dodecyl-β-d-maltoside (DDM) for 1 h at 4 °C. After ultracentrifugation (30 min, 265,000 × *g*, 4 °C) the supernatant was incubated with Ni-Sepharose resin (GE Healthcare) for 1 h at 4 °C. The column was washed with 50 mM Tris HCl, pH 8.0, 300 mM KCl, 0.15% n-decyl-β-d-maltoside (DM), 60 mM Imidazole, pH 8.0, and the protein was eluted with the same buffer containing 500 mM Imidazole. Glt_Tk_ was further purified by size exclusion chromatography on a Superdex 200 10/300 gel-filtration column (GE Healthcare) in 10 mM Hepes KOH, pH 8.0, 100 mM KCl, 0.15% DM.

### Nanodiscs reconstitution and cryo-EM sample preparation

Twenty milligram per milliliter of liposomes containing *E. coli* polar lipids and egg PC (w/w 3:1, Avanti), were solubilized by adding 30 mM DDM followed by 1 min vortexing and 3 h incubation at 4 °C while nutating. Freshly prepared *apo* Glt_Tk,_ cleaved MSP2N2 and solubilized lipids were mixed in 3:5:100 molar ratio (considering a single protein chain) and incubated at 4 °C for 90 min while nutating. To remove the detergent BioBeads (Bio-Rad) were added and the mixture was incubated overnight at 4 °C. After removing BioBeads using a syringe the solution was supplied with 15 mM Imidazole, pH 8.0, and Ni-NTA resin, equilibrated with 50 mM Tris-HCl, pH 8.0, 100 mM KCl to remove empty nanodiscs. After 1 h incubation at 4 °C on a rocking platform resin was washed with 50 mM Tris-HCl, pH 8.0, 300 mM KCl, 30 mM Imidazole, pH 8.0 and nanodiscs were eluted with 500 mM Imidazole in the same buffer. The sample was centrifuged (10 min, 20,000 × *g*, 4 °C) and applied to Superdex 200 10/300 gel-filtration column (GE Healthcare) equilibrated with 20 mM Tris-HCl, pH 8.0, 100 mM NaCl. Fractions with nanodiscs were concentrated using VivaSpin® 500 MWCO 50 kDa concentrators (Sartorius) to 1–4 mg/mL. The sample was supplemented with NaCl to 300 mM (Na^+^-only state) and additionally with l-aspartate (Sigma-Aldrich) at the molar ratios Glt_Tk_: l-aspartate equal to 3:1 for unsaturated state (9 µM of both nanodiscs and l-aspartate) or 1:3 for saturated state (5.6 µM nanodiscs and 50 µM l-aspartate) or with DL-TBOA (4.5 µM nanodiscs and 120 µM DL-TBOA). After mixing the sample was incubated for 30 min on ice. The sample at 0.5–1 mg/mL was applied onto freshly glow-discharged Quantifoil grids (Au R1.2/1.3, 300 mesh) at 22 °C and 100% humidity and plunged-frozen in liquid ethane. The Cryo-EM data were collected using 200 keV Talos Arctica microscope (Thermo Fisher).

### Cryo-EM image processing

Motion correction, CTF estimation, template-based picking, 2D classification, Ab initio volume generation and non-uniform 3D refinement (without symmetry applied if not stated otherwise) were performed using cryoSPARC^47^ (Supplementary Fig. 1). Maps were sharpened using Autosharpen Map procedure in Phenix^48^. The sharpened maps were used for the manual model building using Coot^49^ and refinement of the coordinates was performed in realspace refine module of Phenix^50,51^. Visualization and structure interpretation were carried out in UCSF Chimera^52,53^ and PyMol (Schrödinger, LLC).

For the data obtained using protein in aspartate-free conditions, 1288 micrographs were selected for the processing after motion correction and CTF estimation. The template for particle picking was generated from 100 manually picked particles. Template-based picking identified 1,193,046 particles. Subsequent 2D classification reduced the number of particles to 334,624 and subsequently 180,608 (49%) particles were left in the selected ab initio class. Final non-uniform 3D refinement resulted in a 3.22 Å map, where one protomer was oriented up (*apo* state) and two protomers were inward oriented. Other particles from ab initio volume generation were combined and refined, providing a low-resolution map of 4.82 Å (2 out:1 in).

For the data obtained using protein in substrate unsaturated conditions, 956 micrographs were selected after motion correction and CTF estimation. 348,173 particles were picked and 149,715 particles were left after subsequent 2D classification. Ab initio volume generation provided three classes of 72,313 (48%), 59,666 (40%), and 17,736 (12%) particles. Two big classes were used for non-uniform 3D refinement without applying a symmetry and resulted in maps of 3.38 Å (2 out:1 in) and 3.39 Å (2 in:1 out).

For the data obtained using protein in substrate saturated conditions, 541 micrographs were selected after motion correction and CTF estimation. 444,955 particles were picked and 92,538 particles were left after subsequent 2D classification. Ab initio volume generation provided with one class of 65,762 (71%) particles that was used for non-uniform 3D refinement without applying a symmetry. The resulting map of 4.0 Å resolution revealed symmetrical arrangement of the three protomers, therefore C3 symmetry was applied to generate a final 3.41 Å map.

For the data obtained using protein in TBOA-inhibited conditions, 1,675 micrographs were selected after motion correction and CTF estimation and 578,728 particles were picked. Following 2D classification a set of 228,131 particles was left. After ab initio volume generation one class of 132,917 (58%) particles was chosen for further non-uniform 3D refinement without applying a symmetry. The resulting map of 3.47 Å resolution revealed an asymmetric state with two fully-outward and one inward oriented protomers.

### Reporting summary

Further information on research design is available in the Nature Research Reporting Summary linked to this article.

## Supplementary information

Supplementary Information

Reporting Summary

Peer Review File

## Data Availability

Data supporting the findings of this manuscript are available from the corresponding authors upon reasonable request. A reporting summary for this Article is available as a Supplementary Information file. The source data underlying Fig. 4b is provided as a Source Data file. The cryo-EM density maps of the glutamate transporter homolog Glt_Tk_ in Na^+^-only, unsaturated (2in:1out), unsaturated (2out:1in), saturated conditions and in the presence of TBOA inhibitor have been deposited in the Electron Microscopy Data Bank under accession numbers EMD-10636, EMD-10633, EMD-10634, EMD-10635, EMD-10632, respectively. Coordinates of the corresponding five models have been deposited in the Protein Data Bank under the accession numbers 6XWR, 6XWO, 6XWP, 6XWQ and 6XWN, respectively.

## Source Data

Source Data
