# Supplementary material for: Structural ensemble of a glutamate transporter homologue in lipid nanodisc environment
Source: Nat Commun. 2020 Feb 21;11:998. doi: 10.1038/s41467-020-14834-8 (PMC7035293; doi:10.1038/s41467-020-14834-8)
Supplement: Supplementary file 1 — Supplementary Information [file 41467_2020_14834_MOESM1_ESM.pdf]

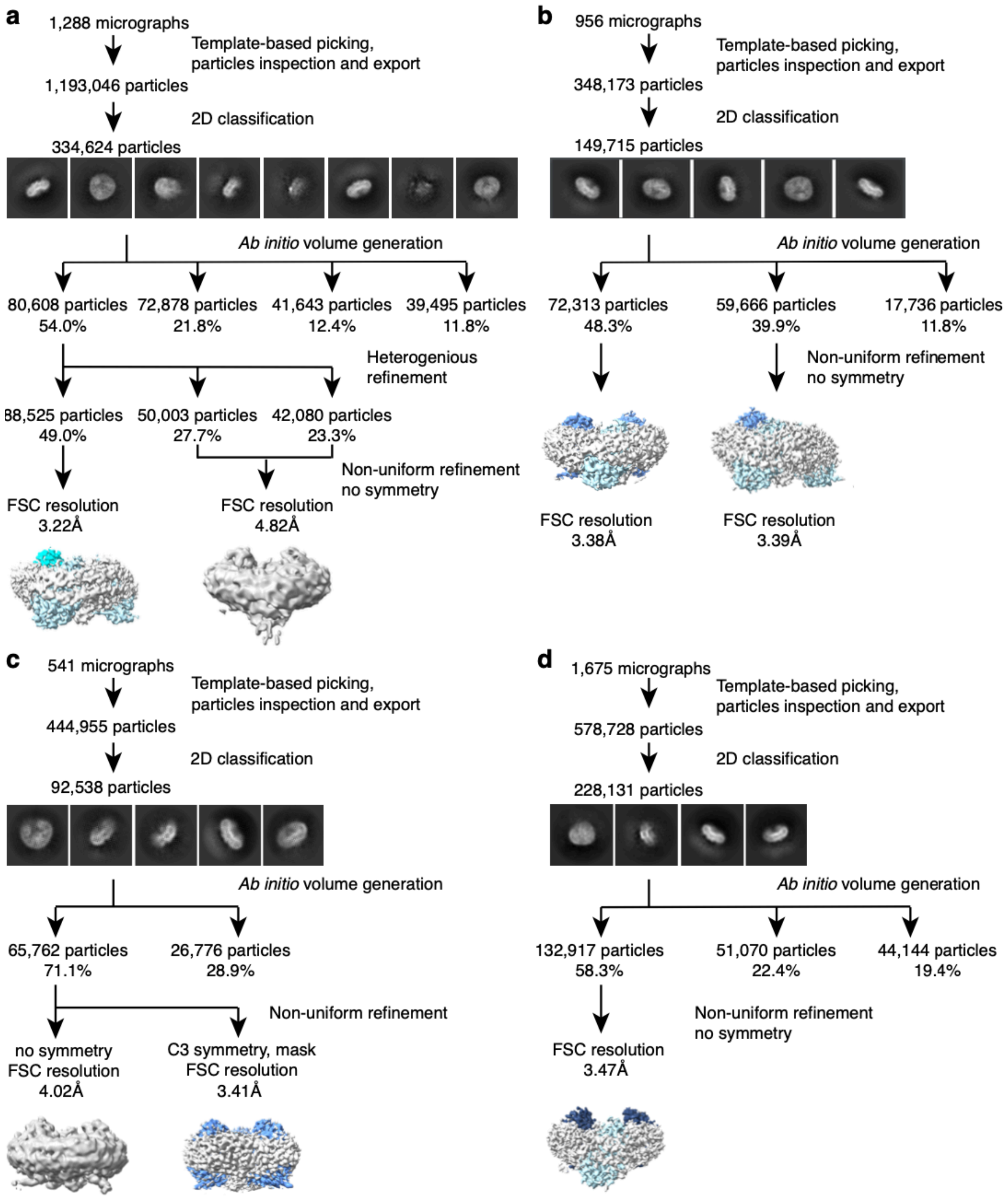

Supplementary Figure 1. Image processing workflow for Glt_{Tk} reconstituted into nanodiscs in **a**. aspartate-free, **b**. unsaturated, **c**. saturated and **d**. TBOA-inhibited conditions. Colors of the transport domains in the final maps are the same as in Fig.1.

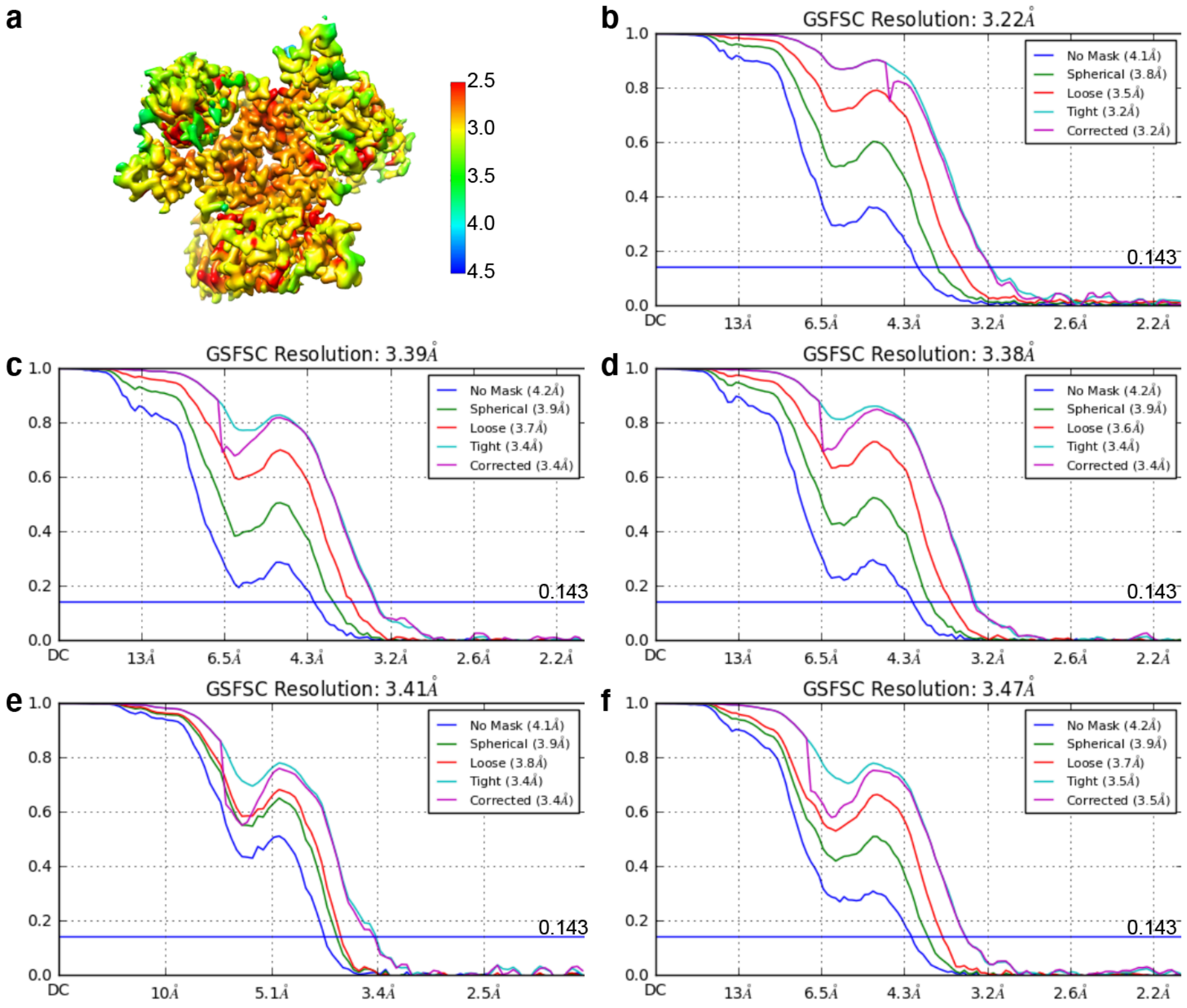

Supplementary Figure 2. Resolution estimation and model validation. **a**. Representation of the final reconstructed map of the Glt_{Tk} in aspartate-free conditions colored by local resolution as estimated in CryoSPARC. Gold standard Fourier shell correlation (GSFSC) curves for Glt_{Tk} in **b**. aspartate-free, **c**. unsaturated 2 in :1 out, **d**. unsaturated 2 out :1 in, **e**. saturated and **f**. TBOA-inhibited conditions.

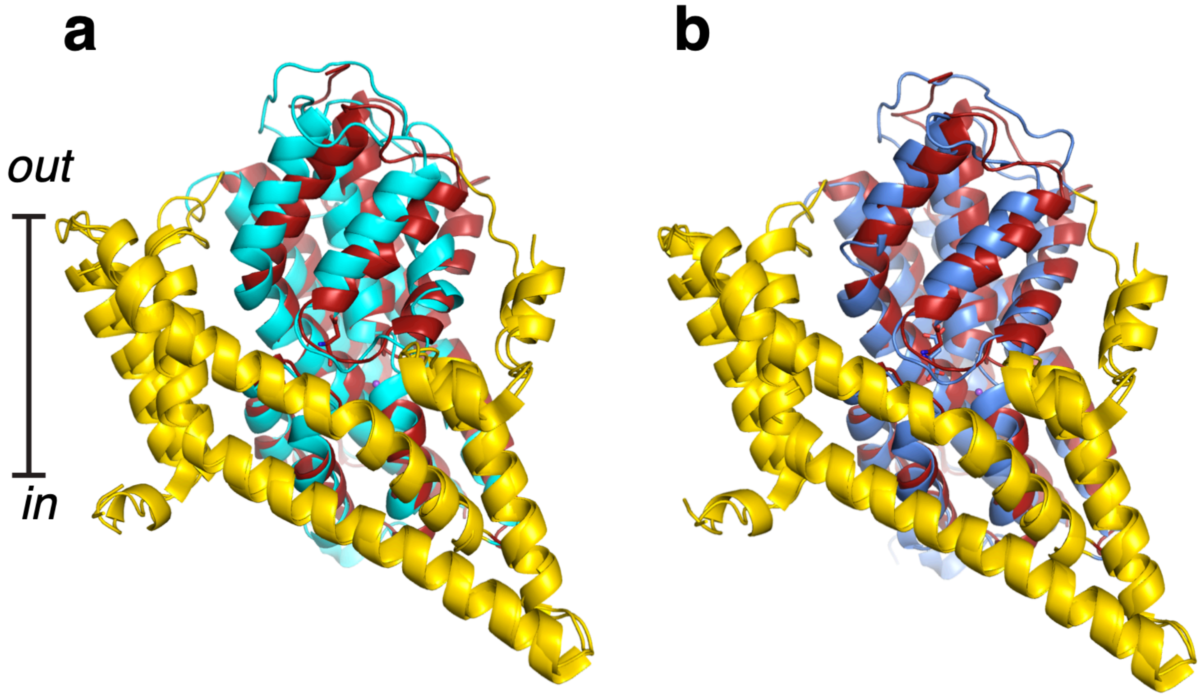

Supplementary Figure 3. Comparison of conformational differences between intermediate outward Glt_{Ph}-*holo* (Asp) (PDB 3V8G, dark red) captured in the crystal structure with the cryo-EM structures obtained here **a.** Glt_{Tk}-*apo* (cyan) and **b.** Glt_{Tk}-*holo* (Asp) (cornflower blue). Scaffold domains are shown in yellow. Superpositions on TM2 and TM5 of the scaffold domain.

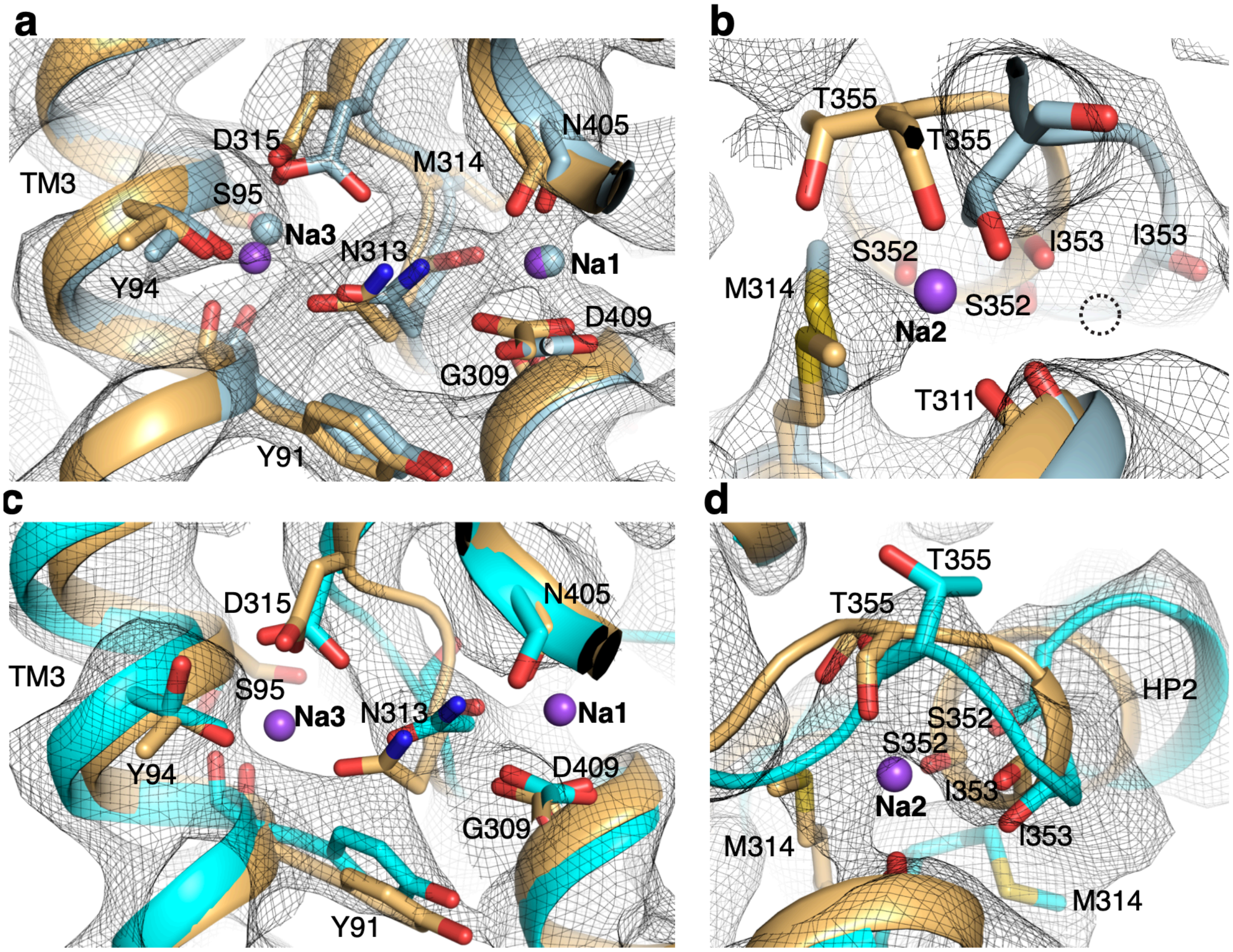

Supplementary Figure 4. Na^+ binding sites. **a.** Modeling of Na^+ ions in the Na1 and Na3 sites of the cryo-EM structure of Na^+-only Glt_{Tk} (light-blue) in comparison with their positions (purple spheres) in the crystal structure of Glt_{Tk}-*holo* (Asp) (PDB 5E9S, light-orange). **b**. Absence of Na^+ in the Na2 site in the cryo-EM structure of Na^+-only Glt_{Tk} (approximate potential position is depicted with a dashed circle with no density). **c.** Absence of density for Na^+ in the Na1 and Na3 sites in the *apo* Glt_{Tk} (cyan). **d.** Absence of the density for Na^+ in the Na2 site in the *apo* Glt_{Tk}. Cryo-EM density is shown as a grey mesh at 3.5σ.

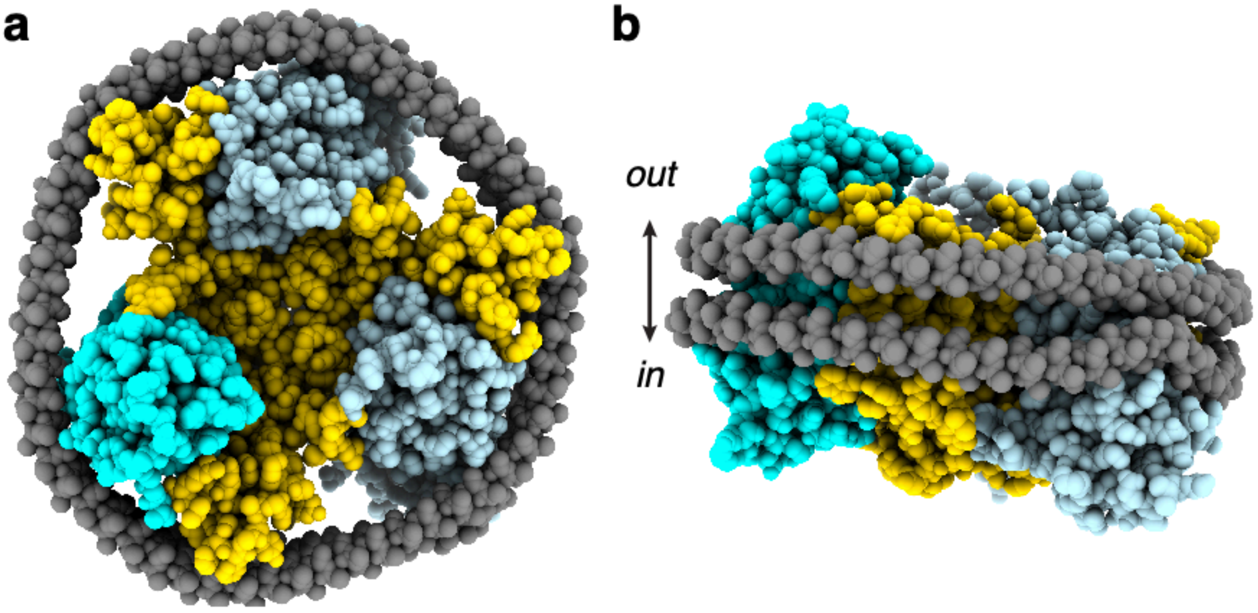

Supplementary Figure 5. Glt_{Tk} and MSP2N2 form nanodiscs of 10-11 nm in diameter and 1.6-2.3 nm width (measured using Cα atoms of the modeled belt protein). **a, b**. Top and side view of the Glt_{Tk} nanodisc model in aspartate-free conditions.

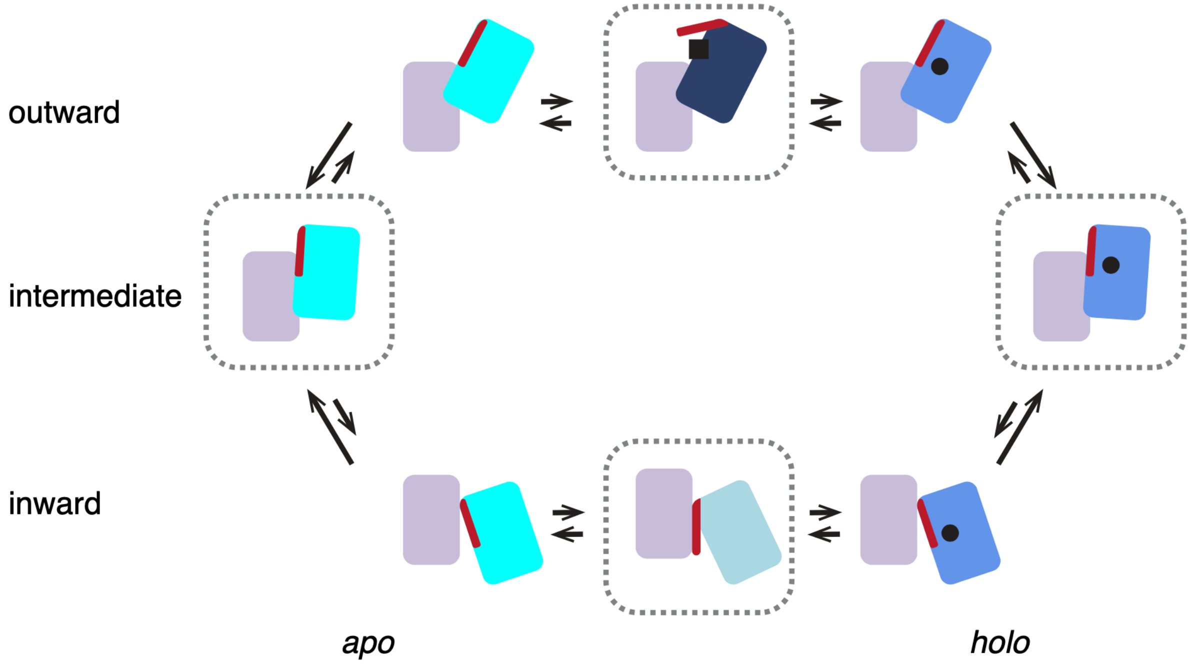

Supplementary Figure 6. Scheme of the transport cycle of glutamate transporters. Four states enclosed in dashed blocks represent structures observed in this work. Glt_{Tk} in both *apo* and *holo* (Asp) states prefers intermediate outward conformations (*apo* and *holo*, respectively) from where it can visit both inward and fully outward conformations. Saturation with Na^+ ions leads to increase of inward open state population. Non-equilibrium conditions *in vivo* (membrane voltage, substrate and Na^+ gradients) will lead to different steady-state distributions over the conformational ensemble. Scaffold domain is shown in grey, the rest of colors and indications are the same as in Figure 1.

Supplementary Table 1. Distances (in Å) between HP1 (Cα S279) and HP2 (Cα V358) in all protomers of the Glt_{Tk} structures:

Chain	Na^+-only	Unsaturated 2in:1out	Unsaturated 2out:1in	Saturated	TBOA
A	4.39	9.65	9.47	4.92	9.09
B	9.10	9.09	5.19	4.88	13.80
C	9.36	4.79	4.88	4.90	14.48

Supplementary Table 2. Distances (in Å) between Cα S279 of HP1 in Glt_{Tk} structures aligned on the scaffold domains:

Distances between Cα S279	Na^+-only	*apo*	Asp	TBOA
Na^+-only	0			
apo	18.3	0		
Asp	18.5	0.5	0	
TBOA	**23.7**	5.7	5.5	0
