# Supplementary material for: Structural ensemble of a glutamate transporter homologue in lipid nanodisc environment
Source: Nat Commun. 2020 Feb 21;11:998. doi: 10.1038/s41467-020-14834-8 (PMC7035293; doi:10.1038/s41467-020-14834-8)
Supplement: Supplementary file 3 — Peer Review File [file 41467_2020_14834_MOESM3_ESM.pdf]

Editorial Note: This manuscript has been previously reviewed at another journal that is not operating a transparent peer review scheme. This document only contains reviewer comments and rebuttal letters for versions considered at *Nature Communications*.

Reviewers' Comments:

Reviewer #1:
Remarks to the Author:
The revision is adequate. This study is a very nice contribution to the field.
